# Comparison of acute non-visual bright light responses in patients with optic nerve disease, glaucoma and healthy controls

**DOI:** 10.1038/srep15185

**Published:** 2015-10-19

**Authors:** M. Münch, L. Léon, S. Collomb, A. Kawasaki

**Affiliations:** 1 Solar Energy and Building Physics Laboratory, Environmental and Civil Engineering Institute, Swiss Federal Institute of Technology, Lausanne, Switzerland; 2 University of Lausanne, Hôpital Ophtalmique Jules-Gonin, Lausanne, Switzerland

## Abstract

This study examined the effect of optic nerve disease, hence retinal ganglion cell loss, on non-visual functions related to melanopsin signalling. Test subjects were patients with bilateral visual loss and optic atrophy from either hereditary optic neuropathy (n = 11) or glaucoma (n = 11). We measured melatonin suppression, subjective sleepiness and cognitive functions in response to bright light exposure in the evening. We also quantified the post-illumination pupil response to a blue light stimulus. All results were compared to age-matched controls (n = 22). Both groups of patients showed similar melatonin suppression when compared to their controls. Greater melatonin suppression was intra-individually correlated to larger post-illumination pupil response in patients and controls. Only the glaucoma patients demonstrated a relative attenuation of their pupil response. In addition, they were sleepier with slower reaction times during nocturnal light exposure. In conclusion, glaucomatous, but not hereditary, optic neuropathy is associated with reduced acute light effects. At mild to moderate stages of disease, this is detected only in the pupil function and not in responses conveyed via the retinohypothalamic tract such as melatonin suppression.

Melanopsin-mediated photoreception within intrinsically photosensitive retinal ganglion cells (ipRGCs) is an irradiance detection system in the eye that operates in parallel with the luminance encoding system of rods and cones^1^,^2^,^3^. The melanopsin system in mammals is involved in several non-visual, light-mediated functions such as regulation of pupil size, circadian photoentrainment, hormonal secretion, sleep regulation, mood and cognitive performance^4^,^5^,^6^,^7^. Axons from ipRGCs project directly to various nuclei in deep brain centers^8^. The most abundant of these monosynaptic projections forms the retinohypothalamic tract (RHT) and synapses at the suprachiasmatic nucleus (SCN) of the hypothalamus^9^,^10^. The SCN is considered the master circadian pacemaker, and the melanopsin system via the RHT is the primary means by which the endogenous biologic clock is entrained to environmental light-dark cycles^1^,^2^. In addition to the circadian effects, light also has acute effects, which occur immediately after onset of light. These include nocturnal suppression of the pineal hormone melatonin^11^, reduced subjective sleepiness, greater attentional vigilance and improved neurobehavioral performance^7^,^12^,^13^.

The ipRGCs also form another important monosynaptic pathway to the brain, the retinotectal tract (RTT) which synapses at the pretectal olivary nuclei of the dorsal midbrain^2^. The RTT is the source of all afferent pupillomotor input from the eye for the pupil light reflex^4^,^14^. While ipRGCs are not required for classical visual functions, they do receive extrinsic input from rods and cones^15^,^16^ which can modulate signalling in the RTT. In humans, rods and cones are suited for detection of rapid changes in light and are primarily responsible for initiating the immediate pupil contraction to an abrupt increase in illumination^17^.

Light at high irradiance (>13 log quanta/cm^2^/s retinal irradiance), particularly in the short wavelength range, strongly activates melanopsin^18^,^19^. In the absence of rod and cone function, the pupil in mammals (rodents and primates) and humans can still react to light via intrinsic, melanopsin-mediated photoreception of ipRGCs^4^,^20^,^21^. On pupillographic recordings in macaque monkeys whose rod and cone activity has been pharmacologically blocked, the distinctive feature of melanopsin to the pupil response is a sustained contraction that persists after light offset^18^,^20^,^22^. This behaviour has been termed the post-illumination pupil response, or PIPR^18^,^22^,^23^,^24^,^25^. Despite the relative paucity of ipRGCs (about 3000 per eye in human and non-human primates)^19^,^26^, there is surprising diversity in their anatomic morphology, molecular expression and kinetics of photic response^26^,^27^,^28^,^29^,^30^,^31^. In mice, at least five subtypes of ipRGCs have been identified. While a strict subdivision of labor amongst ipRGC subtypes is not established, there is nascent evidence suggesting differential roles for ipRGC subtypes with M1 subtype primarily dedicated to circadian photoentrainment^32^,^33^.

In animal models of optic nerve injury and in human optic neuropathies, ipRGCs have shown a greater resistance to certain models of ganglion cell injury and death, compared to conventional retinal ganglion cells^34^,^35^,^36^,^37^,^38^,^39^,^40^,^41^,^42^. Several studies have observed that patients with bilateral visual loss due to mitochondrial dysfunction, such as the isolated hereditary optic neuropathies, retain normal pupil light reflexes^39^,^43^,^44^. Other types of ganglion cell death, such as glaucomatous optic neuropathy, do not appear to spare ipRGCs and melanopsin-mediated functions. Patients with moderate-to-advanced glaucoma demonstrate reduced pupil contraction and reduced PIPR, suggesting impaired signalling in the RTT^45^,^46^,^47^. In addition, they have a reduction in the light-induced suppression of nocturnal melatonin secretion and disturbances in sleep quality, implicating impairment of melanopsin signalling in the RHT pathway^48^,^49^,^50^,^51^.

These and other studies have examined the activity of ipRGCs in patients with visual loss from neuroretinal disease by assessing one parameter known to be modulated by the melanopsin system. However, it is not clear if all or only some of the melanopsin-based functions are altered in such patients and if they change with similar magnitude. We hypothesize that the physiologic functions related to acute light responses predominantly regulated by ipRGCs do show similar and proportionate compromise in the event of death or dysfunction of these cells. In this study, we examined the effect of optic nerve disease on the function and relationship of two mainly melanopsin-signalled functions, the pupil response and the suppression of the pineal hormone melatonin in response to bright light exposure in the evening. In addition to assessing the functional capacity of the RHT and RTT simultaneously, we also assessed cognitive parameters which are acutely influenced by bright light exposure, like subjective sleepiness, reaction time and working memory, in order to understand how dysfunction in one tract or both might relate to other downstream correlates of cognition and behaviour.

## Results

### Baseline measures

As projected for the study, differences in visual acuity (VA), mean deviation of the visual field (MD) and mean peripapillary retinal nerve fiber layer thickness (RNFL) between both patient and control groups were significant for all parameters (t-test; p < 0.05; Table 1). The patients having glaucoma (GL) were significantly older than the patients with hereditary optic neuropathy (HON; p < 0.009) and on average, GL patients had better VA than HON patients (p = 0.005). However, loss of visual sensitivity (estimated from visual field MD) and degree of optic atrophy (estimated from RNFL thickness) were comparable between patient groups (p > 0.1). Habitual wake- and bedtimes and sleep durations were not statistically different between groups (p > 0.06). Both patients groups had significantly higher scores on the Pittsburgh Sleep Quality Index (PSQI) than their controls (p < 0.041). Absolute pupil sizes during baseline recordings (i.e. first 10 s in darkness) were similar in both patient and control groups (p > 0.1), and there was no significant difference between left and right eyes (p > 0.1); therefore, both eyes were averaged for pupil analyses.

### Melatonin

**Figure 1:**
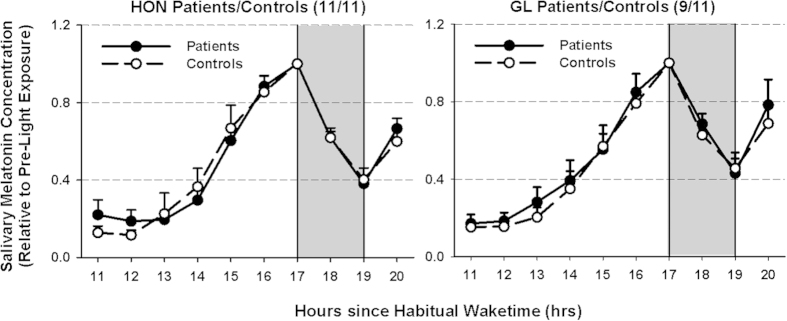
Relative salivary melatonin concentrations for HON patients (left side) and GL patients (right side) and their controls before, during and after 2 hours of light exposure (LE) at night. Values are expressed relative to pre-light exposure melatonin concentrations. In response to light exposure (starting after 17 hours after habitual wake time), salivary melatonin was similarly suppressed in both patient groups (HON and GL) when compared to their controls (p > 0.6). Filled circles and solid lines = patients; open circles and dashed lines = controls. N = 11 in each group except for GL patients (N = 9; mean + SEM; grey areas indicate the light exposure).

Salivary melatonin concentrations were analyzed relative to habitual wake times, with the first sample obtained approximately 11 hours after habitual wake time. Melatonin concentration was plotted as a function of time to ensure rising levels before light exposure (LE). Two GL patients showed no increase in salivary melatonin secretion before LE and were therefore not included in further melatonin analyses. There were no differences in absolute melatonin concentrations (p > 0.5; effect sizes <0.14) between patients and controls across all time points. In order to calculate melatonin suppression, melatonin concentrations during and after LE were expressed (as ratio) relative to the last concentration obtained before LE (Fig. 1). The HON and GL patients showed similar melatonin suppression when compared to their controls (main effect of ‘group’; (HON: F_1,20 _= 0.16; p = 0.70; GL: F_1,18 _= 0.13; p = 0.72; effect sizes: <0.13). Patients and controls showed a significant suppression during light exposure (main effect of ‘time’ without the last sample point after lights off; HON: F_1,20 _= 54.46; and GL: F_1,18 _= 24.82; p < 0.0001).

### Pupil results

**Figure 2:**
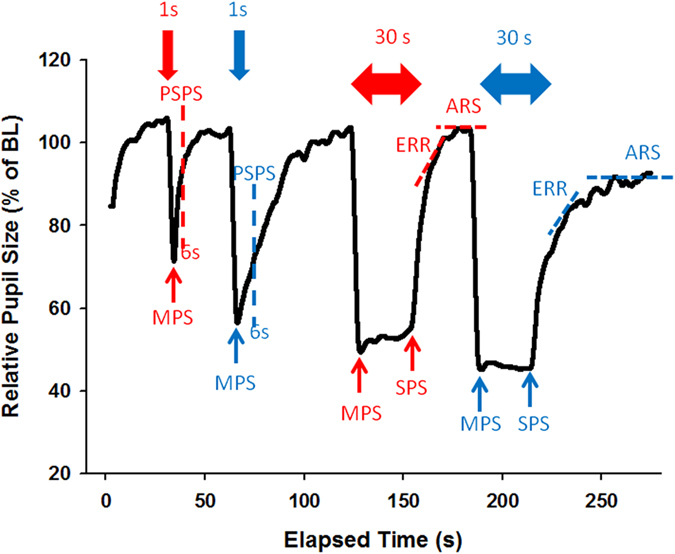
Pupillogram with all metrics and legend with abbreviations. The schematic of the protocol from one recording with the following variables is shown: BL = Baseline pupil size (pupil diameter during the first 10 s of recording in darkness = 100%). Pupil size was expressed relative to baseline (actual pupil diameter/BL pupil diameter*100). MPS = Minimum Pupil size during 1 s and 30 s light stimuli (red and blue); PSPS = Post-Stimulus pupil size at 6 s after 1 s stimulus offset (red and blue); SPS = sustained pupil size; ERR = Exponential redilation rate after 30 s stimulus offset (%−s); ARS = Asymptotic re-dilation size after 30 s light blue and red light stimuli. The bold colored arrows at the top indicate the 1 s red and blue light stimuli as well as the 30 s red and blue light stimuli.

The following abbreviations are used to express pupillary size (diameter) and various aspects of the light response. The reader is referred to the supplement for full description of these pupil parameters. MPS = minimum pupil size, SPS = sustained pupil size, PSPS=post-stimulus pupil size, ERR = exponential re-dilation rate and ARS = asymptotic re-dilation size (ARS), see Fig. 2. SPS, PSPS, ERR and ARS are parameters which describe various aspects of pupillary dynamics in the post-illumination phase. In this study, the main measure of PIPR is the post-stimulus pupil size at 6 seconds from light offset (PSPS).

The first and second pupil recordings, both performed in the same constant dim light (DL) condition, were averaged to obtain a single pre-light exposure (=pre-LE) pupil recording, and this was compared to the pupil recording obtained after 2 hr of bright light exposure (=post-LE). The absolute baseline pupil size was not different between pre-LE and post-LE recordings (p > 0.07). Overall, there were significant differences between red and blue light stimuli for all pupil measures analyzed, except for ARS such that after red light, the pupil tended to be less constricted and to re-dilate faster (=larger MPS, SPS and PSPS; main effect of ‘color’; separately tested for each of the four subgroups; F_1,10 _> 16.9; p < 0.003).

**Figure 3:**
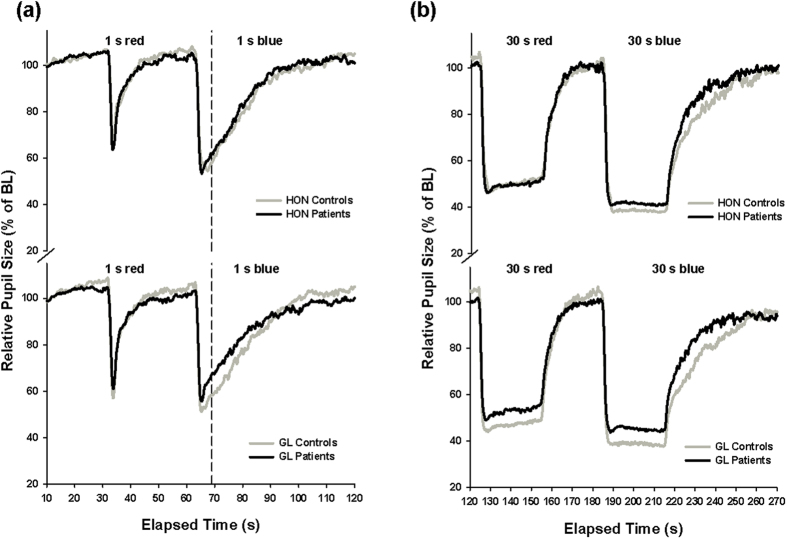
(**a**,**b**) Averaged (from three recordings) pupil tracings for the 1 s (**a**) and 30 s (**b**) red and blue light stimuli for HON patients (upper graphs) and GL patients (lower graphs) and their controls (black lines = patients; N = 11/11; grey lines = controls; N = 11/11). The vertical dashed line indicates the approximate pupil size 6s after light termination (=PSPS). Significant differences were observed between GL patients and controls but not between HON patients and controls except for the minimum pupil size (MPS) during 30 s (p < 0.05). For more results, see Table 2.

For red light stimuli, the MPS after 1 s and 30 s was smaller in HON patients and controls before bright light [main effect of pre-LE vs. post-LE; (F_1,20 _> 11.0; p < 0.003; Table 2, top]. There were no group differences for any of the red light stimuli between both patient groups and controls (F_1,20 _< 3.2; p > 0.08; Table 2 top; Fig. 3), except for a larger MPS and SPS (=less constricted pupils) after 30 s for the GL patients when compared to controls (F_1,20 _> 8.5; p < 0.009). For all patients and controls, the PSPS following red light in the pre-LE recording was significantly larger (=less constricted pupils) than that of the post-LE recording (main effect of pre-LE vs. post-LE; (F_1,20 _> 6.9; p < 0.02; Table 2, top). The ARS was larger for GL patients and controls before (pre-LE) than after bright light (post-LE; F_1,20 _= 12.2; p < 0.02).

Following blue light stimuli, GL patients had significantly larger MPS (i.e. less constricted pupils during 1 s and 30 s blue light stimuli; F_1,20 _> 5.7; p < 0.03), larger SPS (F_1,20 _= 6.2; p = 0.002) and PSPS (after 1 s blue light ; F_1,20 _= 9.2; p = 0.007), smaller ARS (F_1,20_=7.3; p=0.01) and faster ERR after 30 s blue light stimuli (F_1,20_=6.3; p = 0.02) compared to GL controls (main effect of ‘group’; Table 2, bottom). However, there were no group differences in these variables between HON patients and their controls (F_1,20 _< 1.99; p > 0.17), except for the MPS which was larger in HON patients than controls p = 0.041). In both groups (GL and HON), PSPS and MPS (after 1 s) were larger in the post-LE than the pre-LE recording (F_1,20 _> 5.1; p < 0.03; Table 2 bottom). Taken together, between patents and controls, only GL (but not HON) patients showed overall significantly larger PSPS, i.e., less persistent pupil contraction than their controls (p < 0.05) after blue light stimuli.

**Figure 4:**
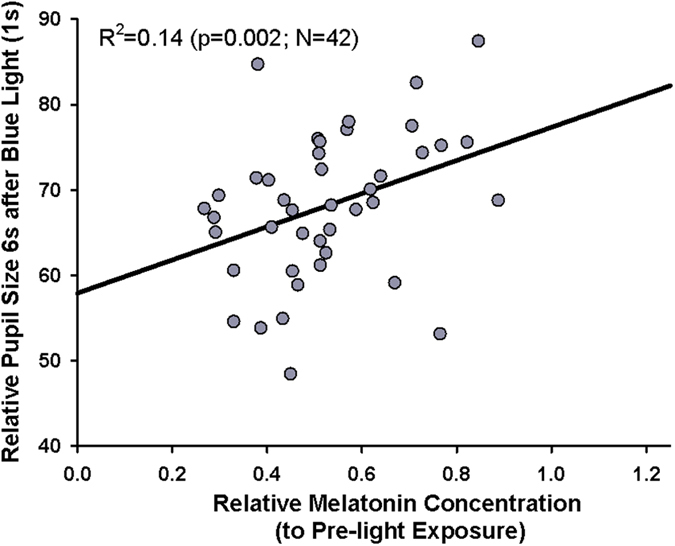
Spearman Correlation between post-stimulus pupil size in response to 1 s blue light (relative to baseline) and mean salivary melatonin concentration (relative to pre-light exposure). Smaller melatonin concentrations indicate greater melatonin suppression; N = 42 (grey circles). The black line shows the regression line (Correlation R^2 ^= 0.14; p = 0.002).

Lastly, we found that correlation of melatonin suppression with relative pupil size after blue light stimuli showed a significant positive association such that individuals who had greater melatonin suppression (i.e. lower salivary concentrations) also had greater post-stimulus pupil constrictions, i.e., had smaller PSPS (R^2 ^= 0.14; p = 0.002; N = 42; Fig. 4).

### Subjective Sleepiness

**Figure 5:**
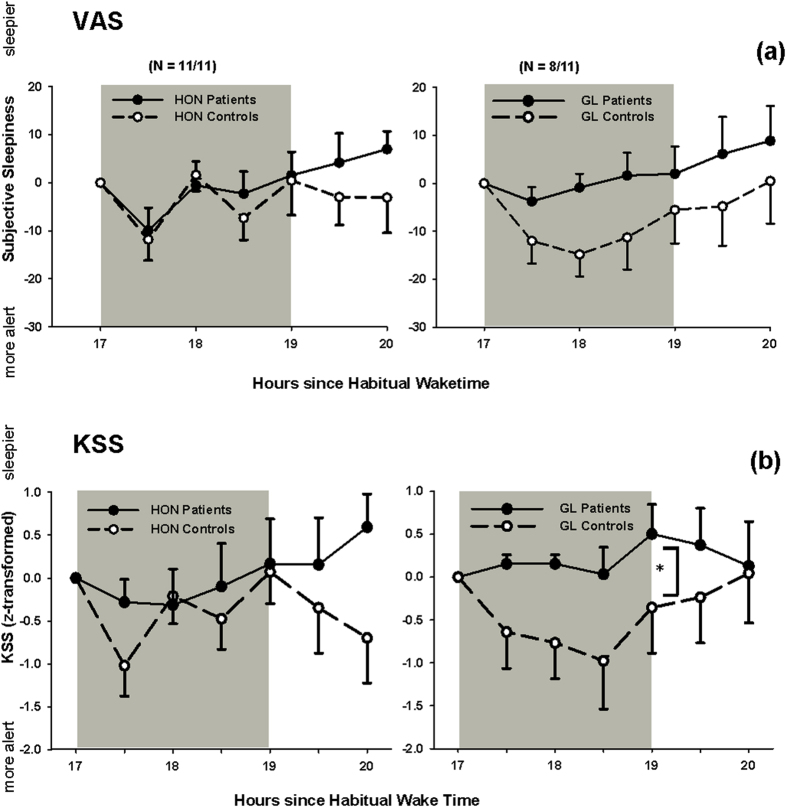
(**a,b**) Subjective sleepiness in patients and controls during and after light exposure assessed from (**a).** Visual analogue scales (VAS; difference to pre-light exposure) and (**b).** Karolinska sleepiness scale (KSS; z-transformed data) in patients (filled circles, solid lines) and controls (open circles, dashed lines). HON patients and their controls are shown on the left side; GL patients and their controls are shown on the right side. From the VAS, HON patients and controls acutely responded to light exposure (p < 0.05; main effect of time), but there was no significant difference in sleepiness between HON and GL patients and controls (means + or − SEM; grey area = light exposure) during and after LE. From the KSS, GL patients became significantly sleepier during LE compared to controls. HON patients respond similarly as their controls.

During the course of the evening both HON and GL groups and their controls became sleepier (HON: F_10,63 _= 4.4 and GL: F_10,53 _= 2.32, respectively; p < 0.02; main effect of ‘time’). There was no absolute difference of subjective sleepiness between both patients groups and their controls across all measurements (p > 0.4; effect sizes <0.34). During the light exposure, there was a significant reduction of subjective sleepiness in both control groups (main effect of ‘time’; p < 0.02; Fig. 5a). There was, however, no main difference between GL or HON patients compared to controls (p > 0.2; effect sizes = 0.65 for GL and effect size = 0.09 for HON). The VAS scale was highly correlated to the KSS (R^2 ^= 0.64, p < 0.05). By analysing the KSS we found that GL patients became significantly sleepier during LE than their controls (main effect of ‘group’; F_1,16 _= 7.3, p = 0.016; effect size = 0.43; Fig. 5b) and the co-variate ‘age’ was also significant (p = 0.0014), but there was no significant difference for the HON patients and controls (p=0.7; effect size=0.42; Fig. 5b). There was a significant interaction for the HON and GL controls with both VAS and KSS (VAS: F_3,30 _= 4.36; p=0.012; KSS: F_3,28 _= 3.29; p = 0.035) during (and after) light exposure and post-hoc analysis (Tukey-Rank Tests adjusted for multiple comparisons) showed that despite an overall reduction of sleepiness especially during the first hour of bright light, HON controls became again sleepier after this first hour (p = 0.02), without any other differences between the two control groups.

### Psychomotor Vigilance Test (PVT)

**Figure 6:**
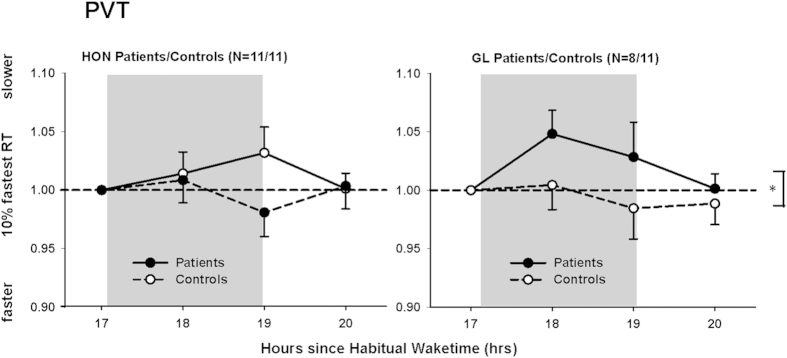
Changes in the 10% fastest reaction times (ms) in the Psychomotor Vigilance Test (PVT) during and after light exposure for HON patients (left side) and GL patients (right side) and their controls. The 10% fastest reaction times (ms) were similar for HON patients and controls (relative to pre-light exposure), but GL patients were significantly slower than their controls in response to bright LE (N = 11 in each group except for GL patients: N = 8; filled circles = patients; open circles = controls; *<0.05). Grey bars indicate constant bright light exposure (means + or – SEM).

The absolute median reaction times (RT) in the Psychomotor Vigilance Test (PVT), the 10% slowest and 10% fastest percentile) revealed no difference between HON and GL patients and their controls (HON: F_1,10 _< 0.73; p > 0.43; effect sizes <0.25; HON patients: 246.0 ± 31 ms; HON controls: 250.6 ± 45 ms; means for all tests ± SD; GL: F_1,7 _< 3.0; p > 0.13; effect sizes <0.15; GL patients 268.8 ± 34 ms: GL controls: 239.4 ± 29 ms; means for all tests ± SD). There were no significant differences between HON and GL patients and their controls for lapses (i.e. RTs >500 ms; p > 0.15; Mann-Whitney U Test). For both patient groups and their controls there was no effect of the factor ‘time’ or any interaction between the factors ‘group x time’ (p > 0.11). When we compared median RT during LE relative to pre-LE (ratios), HON and GL patients showed median RT that was similar to their controls (HON: p = 0.76; effect size = 0.37; GL: p = 0.22; effect size = 0.32), and there were also similar results for the slowest 10% RT in HON and GL patients and their controls (p > 0.29; effect sizes <0.51). For the fastest 10% RT, only GL patients were significantly slower during LE than their controls (F_1,5 _= 14.5; p = 0.013; effect size=0.66 Fig. 6); for HON patients and their controls there was no difference (F_1,5 _= 0.11; p = 0.75; effect size = 0.43 Fig. 6). Unexpectedly, HON controls were significantly slower than GL controls during LE until 1h after LE (relative to pre-light; main effect of ‘group’; F_1,4 _= 58.95; p = 0.002) and the co-variate age also became significant (p = 0.005); but there was no difference between the two patient groups (F_1,2 _= 0.36; p = 0.61).

### Auditory n-back task

The auditory n-back test showed no overall difference in accuracy and reaction time (RT) of the 0-back within both patient groups and their controls (p > 0.2; effect sizes <0.36), indicating that all participants responded properly in pressing a key when no working memory task was involved. There were no differences between the two control groups for any test but in the 2-back test, the controls of the HON group showed significantly better performance than HON patients (main effect of ‘group’; F_1,5 _= 7.4; p = 0.042; effect size = 0.80). There was no difference in accuracy between the GL patients and controls (F_1,5 _= 1.9; p = 0.2; effect size = 0.43). In the 3-back test, HON controls performed again more accurately than HON patients (F_1,5 _= 6.95; p = 0.046; effect size = 1.08) and this was also a trend for the GL control group (F_1,5 _= 4.3; p = 0.097; effect size = 0.72). HON patients and controls became worse in the course of the study in the 2- and 3-back test and had lower accuracy after LE than at the beginning of the study (2-back: F_4,78 _= 2.64; p = 0.04; 3-back: F_4,78 _= 4.28; p = 0.0035; main effect of ‘time’). Besides the above mentioned differences between groups, there were no specific improvements during light exposure for the 2- or 3-back in any of the groups, when the last session after LE was compared to the pre-LE session (p > 0.6).

## Discussion

We aimed at assessing two physiologic functions driven by two different but mainly melanopsin-dependent pathways, i.e., melatonin suppression and the pupil light response (PLR), in visually impaired patients with glaucoma (GL), and with hereditary optic nerve disease (HON). The results were compared to healthy age-matched controls.

Our results on melatonin suppression for HON patients agree with results from another study^40^, such that HON patients and controls responded with similar melatonin suppression to nocturnal light exposure. This preservation of RHT function is thought to be due to selective sparing of ipRGCs in this particular disorder in which a mitochondrial gene mutation is related to retinal ganglion cell death^40^. However, results on melatonin suppression from our GL patient group did not corroborate findings from other studies^48^,^49^. One study of 9 glaucoma patients demonstrated attenuated melatonin suppression under bright (600 lx) and shorter (60 min) light exposure^48^, whereas in our GL patients, melatonin suppression to bright light (4000 lx) was similar to healthy age-matched controls. How could these differences be interpreted? One simple reason may be differences in cohort characteristics and methodology. Our glaucoma patients were younger with less severe disease. In addition, we ensured the timing of melatonin secretion by use of hourly melatonin sampling over a 10 hour period of extended wakefulness in entrained patients, as opposed to a single pre-light and post-light melatonin sample at the fixed time of the night. An alternative explanation may lie in the light intensity such that with lower illuminance, we might have observed distinct differences between GL patients and controls as hypothesized.

In a rodent model of glaucoma, it has been shown that animals with binocularly induced chronic high intraocular pressure took significantly longer than control animals to entrain to light-dark cycles at low illuminances (1–10 lx) but not at higher illuminances (10–100 lx), even though all animals were able to entrain^51^. From this animal model of glaucoma arises evidence of an attenuated circadian response that is evident only at lower light intensities. We may assume that, in our study, overall photic integration of bright light intensity (4000 lx) and a 2 h exposure duration led to saturation for melatonin suppression. Perhaps at lower intensities (or shorter exposure duration), our GL patients may have demonstrated an attenuated suppression of nocturnal melatonin. In addition, the stage of disease was mild to moderate for GL patients in our study. In the previously mentioned study, the GL patients had more advanced disease and this might translate into greater loss of ipRGCs^48^.

The dim light pupil responses to red and blue light showed expected results. The immediate pupil constriction to blue light was greater than that to an equivalent red light. This is a consistent finding, previously reported by us and others^23^,^24^,^52^,^53^, which is presumably and in part due to a greater participation of rods to the immediate pupil constriction to abrupt light onset. After termination of the blue light, the pupil tended to remain contracted, whereas after termination of the red light, the pupil re-dilated quickly and almost reached its baseline size within 6 seconds. Several studies have shown that the spectral sensitivity of the post-illumination pupil response (PIPR) matches that of melanopsin pigment^18^,^22^,^25^ and thus we consider persistently small pupils following blue light offset in this study to be a marker of melanopsin contribution to the pupil light reflex. The post-stimulus pupil size (PSPS) was not affected in the HON group but glaucoma patients had pupils that were less able to sustain contraction following light offset (larger PSPS). Indeed, all parameters of the PIPR were significantly reduced in glaucoma patients. While there were differences in pre-LE and post-LE pupil responses for both red and blue lights, such changes most likely relate to light adaptation effects from all photoreceptive components, enabled via dopaminergic amacrine cells^16^. Other studies have also shown a loss of the PIPR in patients with moderate to advanced glaucoma^45^,^46^,^47^,^54^,^55^. Our study demonstrates that potential ipRGC dysfunction and thus reduced melanopsin activity occurs even in earlier stages of this disease. Our glaucoma patients had only moderate deficits on visual field testing and visual acuity. In contrast, HON patients with similar degree of visual loss but of different pathophysiology, i.e. inherited mitochondrial dysfunction, did not demonstrate any reduction of melanopsin activity as determined from PIPR analysis using 1 s or 30 s of light stimulation. Relative resistance of ipRGCs to mitochondrial dysfunction and cell death has been demonstrated histopathologically in patients with HON and is generally cited as the reason for the preservation of melanopsin-mediated light functions in these patients^38^,^39^,^56^.

We had hypothesized that both circadian and pupillary function (mediated via the RHT and RTT of ipRGCs) would be similarly and proportionately affected by ocular disease causing retinal ganglion cell loss, as a relationship between melatonin and pupil over 24 hours has been reported in previous works in humans^53^,^57^. In the current study, there is indeed a weak but significant positive correlation between the functions mediated by these two tracts. In other words, a greater ability to suppress nocturnal melatonin in response to acute bright light was correlated with a greater ability to keep the pupils contracted after light offset in the same individual. Thus it is not surprising that patients with HON, a disease believed to spare ipRGCs, had no loss in either RHT or RTT function.

To be fair, the correlation was weak (R^2 ^= 0.14) and this may indicate potential sources of variance on melatonin secretion and pupillary contraction. These include central modulating influences at the multiple synaptic sites of the two pathways. Since we did not find statistical differences in melatonin suppression between both control groups and their patients but significant differences in pupil responses, the intra-individual correlation may be questionable. Perhaps melatonin secretion and the pupil light reflex in response to light are functionally different processes and this may be the basis for the unexpected finding that glaucoma patients in this study showed a relative loss of the pupil function but not the melatonin function. In contrast, in more advanced stages of disease, patients with glaucoma have shown loss of melatonin suppression^48^. So why might dissociation in the functional activity of RHT vs. RTT manifest in earlier stages of disease?

One possible reason might be a differential number of ipRGC projections in the RHT compared to the RTT, and this may relate to a differential sensitivity between melatonin suppression and pupil light reflex for detection of dysfunction. If the RHT has a greater number of axons, as indicated in an animal model of glaucoma^51^,^55^, there may be enough redundancy in the circadian system such that mild-to-moderate optic atrophy from glaucoma does not yet affect melatonin responses whereas the RTT and pupillary function might be more susceptible to early loss of ipRGCs.

Another reason may be related to ipRGC subtypes. Despite their scarce numbers, there is surprising diversity in their anatomic morphology, molecular expression and kinetics of photic response^26^,^27^,^28^,^29^,^30^,^31^. In mice, at least 5 subypes of ipRGCs have been identified. The two most populous are M1 and M2 subtypes, found in fairly equivalent proportions^27^,^58^,^59^, and display different photic responses. The M1 subtype responds to light mostly via melanopsin-based photoreception^28^,^29^ whereas M2 subtype generate mostly extrinsic synaptically-driven photoresponses. In addition, there is diversity in the central projections of ipRGCs. In the RHT, light input from the M1 subtype of ipRGCs dominates whereas M2 input may be slightly more favoured in the RTT^28^,^58^,^60^. If M2 subtype of ipRGCs is more susceptible to glaucoma, or alternatively if M1 subtype is a more robust subtype, then loss of pupillary function may be evident earlier in disease compared to the RHT function which may remain spared until more advanced cell death occurs.

A third reason may be simply that the RHT and RTT do not synapse directly at the efferent nuclei for melatonin secretion and pupil light reflex. The RHT and RTT serve as a direct source of retinal light information to the suprachiasmatic and the pretectal olivary nuclei, which are the main integrating nuclei for circadian rhythm and pupil light reflex, respectively. These nuclei also receive various other supranuclear inputs which modulate their signaling through multisynaptic pathways to regulate for example melatonin secretion via the pineal gland and to initiate pupillary constriction via third crania nerve. Even if glaucoma does disrupt signaling through both the RHT and RTT, perhaps there are more central influences or adaptive mechanisms aimed to maintain melatonin secretion at normal functioning.

Attenuation of other non-visual functions in patients with ipRGC cell loss such as in GL patients were shown by lessening of acute alerting effects to bright light exposure on subjective sleepiness and reaction times in the PVT. This is an indication that other central influences on alerting and cognitive functions were selectively impaired in patients with ipRGC cell losses, as recently shown also on sleep with lower sleep efficiency in glaucoma patients than healthy controls^54^. We were not able to find acute light effects in one of the two patient-control groups to an auditory working memory test. It seems that patients, especially HON patients performed overall worse than their controls which possibly indicates that either the test was too difficult and/or patients were altogether too sleepy. Additionally, the small sample size of this study may have precluded finding an effect.

To summarize we found preserved melatonin suppression in GL and HON patients, but only GL patients had larger PSPS (reduced PIPR) when compared to their controls. In glaucoma, this dissociation of disease effect on melanopsin-mediated functions may arise from several factors including relatively early disease state, selective impact or sensitivity amongst ipRGC subtypes to disease or asymmetric influence of central modulating inputs on the pupil and circadian response. In addition, during bright light exposure, GL patients were sleepier with slower reaction times compared to controls suggesting that there may be an influence of reduced ipRGC signaling on cognitive and behavioural functions.

## Methods

### Study design

Each participant came to the eye hospital once during daytime for baseline ophthalmological examination. At this visit, participants were also trained for the cognitive testing, underwent a baseline pupil recording and received instructions for use of the activity monitor. Thereafter, participants were asked to maintain a regular sleep wake-cycle during one week prior to the study. This included moderate consumption of caffeine and alcoholic beverages and a sleep schedule of approximately 8 hours in bed at the same times each night (within a range of 30 minutes). Compliance with the latter was verified by wearing a wrist activity monitor (Actiwatch L, Respironics AG, Schweiz) and maintenance of a sleep diary.

At the end of the entrainment period, participants were asked to come to the photo biological laboratory at the Swiss Federal Institute of Lausanne during evening time for the study testing. Each participant was individually tested on a different night. Compliance for medication and drug absence was verified with a urinary toxicological screen before the night testing. The study testing started 10 hours after habitual wake time and lasted for 10 hours. Room lighting was maintained at a constant dim illumination (<6 lx). Participants were able to talk and to listen to music or audio books. Portable electronic appliances with screens were not permitted due to the additional light exposure. Small meals and water were provided on a scheduled basis. Throughout the 10 hours in the laboratory, the participants were regularly asked to rate their level of sleepiness. Throughout the evening, salivary samples were collected in a plastic cup every hour; the first sample was obtained approximately 11 hours after habitual wake time. Every 60 to 120 minutes the participants performed two auditory cognitive performance tests.

For the first 7 hours, the participant remained seated in dim light conditions (<6 lx). After 7 hours, the participant was exposed to 2 h of polychromatic white bright light and then sat in DL again for the last hour of the study. The experimental 2 hours of polychromatic bright light exposure (LE) for the melatonin suppression test started 17 hours after their habitual wake time. For this purpose, the participant was seated in front of a large light screen (1.57 × 1.22 m) at 0.5 m distance and which contained fluorescent tubes [34 FL tubes; TLD 50W/94 HF; (Philips) see supplemental Figure S1 for spectral characteristics of the light source]. Participants were instructed to keep their eyes open and to look towards the screen. The illuminance at the eye level in a vertical direction was set to be 4000 lx and was verified for each participant during LE. Two times before, and immediately after LE, a PLR was recorded on both eyes. Every 60–120 minutes the participants performed two auditory-based cognitive performance tests (see supplemental document). Compliance with all study procedures was verified by a trained person who was present throughout the study in the same room. All study participants provided oral and written informed consent for study participation. Study procedures were reviewed and approved by the local ethical commission (Commission d’Ethique de Recherche sur l’être humain de Canton de Vaud, Switzerland) and were in accordance with the Declaration of Helsinki. Detailed information on the methods as described above, the statistics, the screening procedures and inclusion criteria for patients with hereditary optic nerve disease (n = 11) and glaucoma (n = 11); as well as age-matched controls (n = 22), can be found in the supplemental document (p S1–S13).

## Additional Information

**How to cite this article**: Münch, M. *et al.* Comparison of acute non-visual bright light responses in patients with optic nerve disease, glaucoma and healthy controls. *Sci. Rep.* **5**, 15185; doi: 10.1038/srep15185 (2015).

## Supplementary Material

Supplementary Information

## Figures and Tables

**Table 1:** Demographics of patients with optic nerve disease (hereditary optic neuropathy HON and glaucoma GL) and their controls.

Group	Sex	Age (yrs)	WT	HO	PSQI	BDI	Pupil (mm)	MD	RNFL	VA
HON Patients	4F 7M	39.4# (15.2)	6:49 (0:56)	58.1 (5.7)	4.4* (2.0)	1.7 (1.8)	4.88 (1.65)	7.3* (5.4)	63.9* 10.5)	0.4*# (0.3)
GL Patients	8F 3M	54.1 (7.1)	6:11 (1:15)	62.0 (8.3)	5.5* (3.8)	2.2 (2.0)	4.61 (1.01)	11.4* (6.2)	59.8* (16.5)	0.7* (0.2)
HON Controls	8F 3M	36.2§ (13.2)	7:17 (0:40)	58.6 (9.6)	2.4 (1.9)	0.9 (1.0)	5.15 (1.28)	−0.7 (0.9)	104.5 (7.9)	1.1 (0.1)
GL Controls	7F 4M	54.4 (7.2)	7:02 (0:41)	59.4 (7.1)	2.8 (1.5)	1.6 (1.5)	4.99 (0.79)	–0.6 (0.8)	96.5 (13.1)	1.0 (0.1)

Demographics for GL and HON Patients (Pat) and Controls of both groups; mean (± SD); N = 11 in each group. WT=habitual wake time (clock time); HO = Horne Ostberg; PSQI = Pittsburgh Sleep Quality Index; BDI = Beck Depression Inventory; MD = Mean Deviation of automated perimetry; RNFL = mean peripapillary retinal nerve fiber layer thickness (μm); VA = Visual Acuity.

^*^significant differences (p < 0.05) between patients and their controls (separately for GL and HON).

^#^significant difference between both patient groups (p < 0.05).

^§^significant difference between both control groups (p < 0.05). Absolute pupil sizes are indicated as mean values for all tests per participants and all ophthalmological values (pupil size, MD, RNFL, VA) were averaged for left and right eyes.

**Table 2:** Pupil parameters for patients with optic nerve disease (hereditary optic neuropathy HON and glaucoma GL) and their controls (N = 44).

Red Lights	Controls				Patients			
	Mean Pre-LE	(SD)	Mean % Post-LE	(SD)	Mean Pre-LE	(SD)	Mean Post-LE	(SD)
GL								
MPS (1s)	55.5	(4.8)	56.6	(8.2)	60.9	(6.6)	60.9	(8.1)
MPS (30s)*	40.7	(8.0)	43.5	(3.6)	48.7	(4.9)	49.5	(6.4)
SPS (30s)*	48.2	(5.3)	50.4	(5.2)	55.3	(6.3)	56.5	(7.0)
PSPS (1s)#	92.8	(6.1)	89.7	(8.8)	95.3	(4.2)	90.9	(8.2)
ERR (30s)	−4.78	(0.06)	−4.80	(0.06)	−4.81	(0.08)	−4.87	(0.11)
ARS (30s)#	107.0	(8.3)	104.8	(9.7)	104.7	(5.3)	97.7	(7.8)
HON								
MPS (1s)#	59.2	(4.6)	62.9	(5.2)	57.1	(6.9)	61.0	(6.4)
MPS (30s)#	43.2	(3.0)	47.3	(4.6)	44.1	(4.4)	46.3	(6.7)
SPS (30s)	53.5	(6.0)	54.3	(5.5)	51.2	(4.9)	52.3	(6.2)
PSPS (1s)#	95.5	(4.0)	89.2	(5.4)	94.1	(5.3)	91.6	(4.9)
ERR (30s)	−4.82	(0.10)	−4.82	(0.07)	−4.78	(0.10)	−4.86	(0.15)
ARS (30s)	105.1	(7.4)	100.7	(3.8)	109.1	(17.6)	103.1	(8.6)
Blue Lights								
GL								
MPS (1s)*,#	46.9	(7.5)	50.1	(6.1)	53.7	(4.0)	55.9	(7.2)
MPS (30s)*	36.3	(8.2)	37.9	(4.8)	43.6	(4.0)	43.6	(5.3)
SPS (30s)*	37.6	(7.7)	37.4	(5.1)	45.6	(4.3)	44.2	(5.7)
PSPS (1s)*,#	56.5	(8.4)	64.2	(9.9)	66.4	(7.3)	73.6	(8.9)
ERR (30s)*	−4.67	(0.03)	−4.67	(0.04)	−4.70	(0.07)	−4.75	(0.05)
ARS (30s)*	106.4	(14.0)	104.0	(7.6)	96.9	(13.1)	98.6	(8.6)
HON								
MPS (1s)#	46.1	(2.2)	52.2	(4.4)	48.9	(5.3)	53.1	(5.0)
MPS (30s)*	36.9	(2.8)	37.3	(4.7)	38.6	(3.6)	42.0	(5.8)
SPS (30s)	38.3	(3.5)	38.4	(3.2)	40.7	(4.1)	40.7	(3.8)
PSPS (1s)#	55.6	(4.7)	68.4	(7.6)	62.7	(8.1)	69.0	(8.3)
ERR (30s)	−4.72	(0.10)	−4.69	(0.03)	−4.72	(0.11)	−4.75	(0.10)
ARS (30s)	103.1	(7.6)	104.6	(14.5)	104.3	(13.2)	103.4	(8.3)

Results of the pupillary light reflex (PLR) to red (at the top) and blue light (at the bottom) before (=pre-LE) the 2-hrs of bright light exposure and after constant bright light (post-LE). MPS = minimum pupil size for 1 s and 30 s light stimuli; SPS: sustained pupil size at the end of the 30 s stimulus; PSPS = post-stimulus pupil size after 1 s light stimuli; ERR = exponential re-dilation rate after 30 s stimuli (%-s); ARS = asymptotic re-dilation pupil size for the exponential fitting. All mean values are shown ± SD (in brackets) and for controls left side and patients (right side). Results for glaucoma (GL) patients and controls are shown in the first four rows and results for hereditary optic neuropathy (HON) patients and controls are shown in the lower four rows.

^*^Significant difference between controls and patients within a group (GL or HON).

^#^significant difference between pre-LE and post-LE (p < 0.05; N = 44).
