# Supplementary material for: Comparison of acute non-visual bright light responses in patients with optic nerve disease, glaucoma and healthy controls
Source: Sci Rep. 2015 Oct 19;5:15185. doi: 10.1038/srep15185 (PMC4609937; doi:10.1038/srep15185)

Supplemental File

Comparison of acute non-visual bright light responses in patients with optic nerve disease, glaucoma and healthy controls

M Münch[1, 2], L Léon[3], S Collomb[3], Kawasaki A[3*]

[1] Solar Energy and Building Physics Laboratory, Environmental and Civil Engineering Institute, Swiss Federal Institute of Technology, Lausanne (Switzerland)

[2] Current address: Charité University Medicine Berlin, Institute of Physiology, Sleep Research and Clinical Chronobiology, Berlin (Germany)

[3] University of Lausanne, Hôpital Ophtalmique Jules-Gonin, Lausanne (Switzerland)

Screening Procedures

All participants completed an entrance questionnaire, the Pittsburgh Sleep Quality Index (PSQI), the Horne Ostberg Morningness Eveningness Questionnaire (HO) and the Beck Depression Inventory (BDI). Only non-pregnant women and men over age 18 years who did not cross more than two time zones within the last three months and who did not work on night shifts during the last 12 months were considered for participation in the study. All study participants came to the eye hospital (Hôpital Ophtalmique Jules Gonin, Lausanne, Switzerland) for an interview and underwent a baseline ophthalmologic examination which included best-corrected visual acuity, color vision testing with Ishihara book and non-dilated funduscopy. Visual fields were assessed using threshold automated perimetry of the central 30 degrees (Octopus 101, Interzeag, Bern-Köniz, Switzerland). The macula and peripapillary retinal nerve fiber layer (RNFL) was examined by optical coherence tomography (OCT; Stratus 3000, Carl Zeiss, Meditec, Inc., Dublin, CA).

Ophthalmologic patients

Patients with bilateral visual loss from either chronic open-angle glaucoma or hereditary optic neuropathy (see below) were recruited from the neuro-ophthalmology unit, the glaucoma unit and general eye clinic at the Hopital Ophtalmique Jules-Gonin in Lausanne (Switzerland). Inclusion for the patient group with diagnosis of isolated hereditary optic neuropathy (HON) was based on the following clinical criteria: subnormal vision diagnosed at childhood or young adulthood, evidence of stable or progressive visual dysfunction since then, bilateral and symmetric central visual loss, bilateral optic atrophy, a positive family history of subnormal vision, bilateral optic atrophy and absence of other neurologic deficit. In addition, investigative tests

including electroretinography and neuroimaging were negative for any other cause of optic nerve damage. Inclusion for the patient group with a diagnosis of chronic open angle glaucoma (GL) was previous documentation of elevated intraocular pressure, bilateral visual field defects typical of glaucomatous visual loss without evidence of progression in the preceding year, and optic disc cupping and no other cause of optic nerve disease.

Glaucoma patients previously treated with trabeculectomy were excluded due to potential effect on iris structure and pupillary movement. Patients using topical agents with known effects on pupillary function, e.g. pilocarpine, brimonidine were also excluded. Diabetes and other neurologic deficit, for example hearing loss were exclusionary factors. A total of 1100 medical charts were screened for inclusion in the study. Due to the rigorous exclusionary criteria, only 50 patients were identified as potential study participants and were invited to fill out questionnaires and to undergo an ophthalmological examination. Twenty five of those patients agreed to participate in the study. Two patients did not complete the study due to acute sickness and one patient was excluded retrospectively due to current use of pain pills and alcohol abuse which were not stated at the time of interview. A total of 11 patients with hereditary optic nerve disease and 11 glaucoma patients were included in the final data analysis.

The 11 HON patients were four women and seven men aged 21 to 64 years (39.4 ± 15.2 years; mean ± SD; Table 1). None was taking a centrally acting medication. None of the HON patients was an extreme morning type, and PSQI scores ranged from 1 to 7 (4.4 ± 2.0), with three patients having PSQI scores greater than 5. The BDI was on average 1.7 ± 1.8 and ranged from 0 to 5. In 7 patients, results of gene testing were available from chart review. Three patients had a mutation of mitochondrial DNA associated with Leber hereditary optic neuropathy. In

two patients with a primary point mutation, 1 had double mutation at position 14484 and position 15257 and 1 patient had mutation at position 3460. The third patient had a non-primary mitochondrial DNA mutation. Four patients had genetic analysis for both Leber hereditary optic neuropathy and dominant optic atrophy and in 3, the results were reported as negative whereas the fourth patient had a point mutation on the short arm of chromosome 3 but the specific OPA1 gene mutation that accounts for two-thirds of patients with dominant optic atrophy was not found.

Visual acuity of HON patients ranged from 0.01 to 1.0 (0.4 ± 0.3; for all eyes; mean ± SD), one patient could count fingers at a distance of 2 m - his acuity was 0.01). All HON patients demonstrated bilateral, symmetric central visual field deficits with a mean deviation (MD) ranging from -1.8 to 17.8 db (7.3 ± 5.4 db). All HON patients had bilateral and symmetric optic atrophy; in three patients the pallor appeared confined to the temporal side of the optic disc. The mean peripapillary RNFL was 63.9 ±.10.5 μm (range from 26 to 94 μm).

The GL patient group consisted of eight women and 3 men whose age ranged from 40 to 63 years (54.1 ± 7.1 years; mean ± SD; Table 1). Two patients were extreme morning types (HO scores>70); the PSQI scores ranged from 1 to 11 (mean ± SD: 5.5 ± 3.8) with four scores $\geq$ 5, indicating some sleep related problems in these patients. The BDI was on average 2.1 ± 1.9 and range from 0 to 7. Visual acuity (VA) ranged from 0.05 and 1.0 (0.7 ± 0.2). Mean deviation (MD) ranged from 1.7 db to 24.2 db (11.4 ± 6.2) and mean peripapillary RNFL in the OCT was 59.8 ± 16.5 μm (range: 35 to 95 μm).

Age-matched controls

For the control group, healthy non-smoking volunteers were recruited via flyers in the region of Lausanne (Switzerland). Controls were matched to patients' age (± 3 years). All control participants were without psychiatric, medical or ocular disorders and not taking any prescription or non-prescription medications on a regular basis. For control subjects, the inclusionary criteria from questionnaires included a PSQI to be lower or equal 5 (to exclude any sleep disorders), an HO score between 30 and 70 (to exclude extreme chronotypes) and a BDI less than 10 (to exclude for depression). All age-matched control participants had to have a normal ophthalmologic examination with no evidence of previous or current ocular disease other than refractive error. All controls had visual acuity of 1.0 or better (1.1 ± 0.1) and identified all 13 Ishihara color plates independently with each eye. The visual field of each control was judged to be normal and the MD for all controls ranged from -2.5 to 0.7 db (mean ± SD: -0.7 ± 0.8 db). Similarly, the OCT of controls was read as normal and the peripapillary RNFL measured 100.5 ± 11.3 μm, mean ± SD (range 68 to 125 μm). The HON control group ranged from 19 to 59 years and was composed of eight women, 3 men (age: 36.2 ± 13.2 years). The GL control group ranged from 42 to 63 years, with seven women and four men (54.4 ± 7.2 years). The demographic and ophthalmologic features of the patients and controls are presented in Table 1.

Methods

Salivary melatonin

Salivary samples for melatonin assays were obtained every hour and then immediately stored at 4° C. After study completion, the samples were centrifuged and frozen at -20° C before sending them to an external laboratory for radio-immuno-assays (RIA; Dr. B. Middleton; University of Surrey; Guildford; UK). The inter-assay

coefficients of variance were 12.4% (low) and 8.5% (high). The intra-assay coefficients of variance were 6.9 % (low) and 2.4% (high) with a detection limit of 0.6 pg/ml.

Pupillometry

The computerized pupillography was performed twice under dim light conditions (one hour after the study began and immediately before bright light exposure), and once after the 2 h of bright light exposure during the night. A Color Dome Ganzfeld ERG apparatus (Diagnosys, Lowell, Massachusetts USA) was used to present a full-field 1 s or 30 s light stimulus at preselected spectral bandwidths of 635 ± 20 nm (red light) and 464 ± 26 nm (blue light) to both eyes simultaneously on undilated pupils. The pupil diameter of both eyes was continuously recorded at 60 Hz by a dual channel binocular pupillometer mounted on an eye frame (Arrington Research, Scottsdale, AZ USA). Following 30 s of pupillary recording in total darkness, a 1 s bright red light then a 1 s bright blue light stimulus (equiluminant for photopic sensitivity at 200 cd/m^2 after calibration, which corresponds to 14.9 log $photons/cm^2/s$ for blue and 15.1 log $photons/cm^2/s$ for red light; according to the manufacturer of the Ganzfeld apparatus), was presented. The dark interval after red light was 30 s and the dark interval after blue light was 60 s (to account for the greater persistence of pupillary constriction after the blue light stimulus). The same red and blue light stimuli were repeated by using 30 s duration of light stimulation.

For pupil data from the right and left eye recordings a customized filter was applied to remove artifacts from blinking and eye movements (Microsoft Excel 2002, Visual Basic for Applications V. 6.5). Pupil tracings were then smoothed by a polynomial smoothing function (Savitzky-Golay; Origin Pro v.8.50 SRO). The

baseline pupil size was defined from the averaged size during the first 10 s of recording in darkness. Actual pupil size was divided into baseline pupil size to convert all values to relative pupil size (RPS) in percentage. The immediate pupil response to light stimulation was assessed by the minimum pupil size (MPS) for 1 s and 30 s stimuli (taken as the smallest RPS immediately after light onset); the sustained pupil response to 30 s was the RPS before light offset (sustained pupil size or SPS =averaged RPS of the last one second before light offset). The distinctive pupillographic feature of melanopsin contribution is the persistent pupillary constriction after stimulus light termination. Therefore, in addition to the immediate pupil constriction to 1 s and 30 s of light (=minimal pupil size; MPS), and the sustained pupil constriction at the end of the 30 s stimuli (SPS), we also analyzed the dynamics of pupil recovery from the point of its maximal constriction. For the 1 s light stimulus, we determined the post-stimulus pupil size (PSPS) after 6 s, calculated as the mean RPS between 5.5 s and 6.5 s after light termination [1-3]. For pupil tracings obtained from the 30 s light stimulus, an exponential fitting was applied on smoothed tracings to obtain the post-stimulus recovery curves by using an asymptotic exponential function: $y = a\text{-}b*c^x$ (a=asymptotic maximum, b=response coefficient and c=rate). Post-stimulus pupillary dynamics was assessed from the exponential re-dilation rate (ERR) and asymptomatic re-dilation size (ARS) from the exponential fitting.

There was no statistical difference between left and right eye pupil size ($p>0.27$ patients and $p>0.1$ controls), therefore pupil data from both eyes were averaged in all analyses. This was done to account for any potential differences that might occur from a difference in baseline pupil size, e.g. anisocoria. For two GL patients, eye movement artifacts precluded using data from one eye. A total of 6.4 %

of all the recordings after the 30 s red and blue light stimuli did not converge to an exponential function.

Subjective Sleepiness

Subjective sleepiness was assessed every 30 min by paper versions of the visual analogue scale. On this scale, the participants had to rate their subjective sleepiness on a continuous line of 100 mm length between two extremes (= 0 mm: very alert; 100 mm: extremely sleepy). The Karolinksa Sleepiness scale is also a valid instrument for subjectively assessed sleepiness [4]. It is a distinct 9 –item scale where participants have to indicate by distinct numbers how sleepy they are. The scale goes from ...'not sleepy at all (1 pt) to...very tired, fighting sleep'... (9pts).

Cognitive Performance

Two auditory-based cognitive performance tests were administered. Every hour, participants had to complete the 5-minute version of the Psychomotor Vigilance Task (PVT) [5]. In this task, the participant heard single tones and had to press the space key on the laptop as quickly as possible. A maximum of 50 tones were presented in random intervals. For the analysis, median reaction time (RT) and the 10% fastest and 10% slowest RT per trial were analyzed. Lapses, defined as RT > 500 ms were calculated separately, and RTs < 150 ms (anticipation) were not included in the analysis. The second performance test, the auditory n-back[6] was completed every two hours (five sessions). In this task, participants had to respond to spoken letters by pressing keys for correct or incorrect answers. In the 0-back test, the correct answer was when the participant heard the letter 'K' and pressed 'yes'; in the 2-back test the participant had to press 'yes' when the current letter which was played to the participant was identical with the penultimate one, otherwise the

participant had to press 'no'. In the 3-back test the participant had to press 'yes' if the current letter which was played to the participant was the same as the third last one, otherwise the participant had to press 'no'. The order of the letters was different for each n-back test and each test session; each of the five test sessions contained five 0-, 2- and 3-backs trials in a randomized order and in each trial a total of 30 letters were presented. The entire test lasted approximately 8 minutes. During the daytime screening visit and before the first test session in the evening, the participant was instructed and was trained with a demo-version, where feedback was given. During the test, the participants received no feedback on their performance. The results were analyzed by calculating accuracy as hits minus false alarms for each n-back version separately.

Statistics

Statistical analyses were performed by using the software packages SAS (SAS Institute Inc., Cary, NC, USA; v9.3 and Statistica v9). For single comparisons we applied two-tailed t-tests. For VAS, PVT and n-back tests, three GL patients were excluded from the analysis since they had reported use of sleep pills (two patients) and antihistamines (one patient) on a non-regular basis. Urinary toxicological screen for these three patients was however negative. Salivary melatonin, VAS, PVT and N-back data were analyzed with a mixed linear regression model (proc mixed) with the fixed factors='group' (patients vs. controls; separate for HON and GL patients); and the repeated factor 'time' (=time bins since study start; i.e. 10 hours for absolute and 3 hours on relative values since the beginning of LE), if not otherwise stated in the text. For the lapses in the PVT a non-parametric test (Mann-Whitney U Test) was used. The age was included as covariate in the analysis of cognitive performance tests (PVT and n-back) and subjective sleepiness (KSS and VAS). The analyses

were performed on log- or square root transformed data if the data was not normally distributed. VAS comparisons between groups were analyzed with relative data (differences to pre-light exposure). For KSS analyses the absolute data were z-transformed and plotted as difference relative to pre-light exposure. All p-values were adjusted for multiple comparisons with the Tukey-Kramer test and the degrees of freedom were adjusted (after Kenward-Rogers). The effect sizes (Cohen's d) were indicated for the melatonin and subjective sleepiness and PVT results in the text and were plotted for the pupil results in supplemental material (Figure S2; d=2 small effect, d=0.5 medium effect and d=0.8 large effect). To examine the relationship between the PSPS and relative melatonin suppression a Spearman rank correlation analysis was performed.

References (for supplement)

1 Park, J. *et al.* Toward a clinical protocol for assessing rod, cone, and melanopsin contributions to the human pupil response. *Invest Ophthalmol Vis Sci* **52**, 6624-6635 (2011).

2 Münch, M., Léon, L., Crippa, S. & Kawasaki, A. Circadian and wake-dependent effects on the pupil light reflex in response to narrow-bandwidth light pulses. *Invest Ophthalmol Vis Sci* **53**, 4546-4555 (2012).

3 Alpern, M. & Campbell, F. The spectral sensitivity of the consensual light reflex. *J Physiol* **164**, 478-507 (1962).

4 Åkerstedt, T. & Gillberg, M. Subjective and objective sleepiness in the active individual. *Intern J Neuroscience* **52**, 29-37 (1990).

5 Dinges, D. F. & Powell, J. W. Microcomputer analyses of performance on a portable, simple visual RT task during sustained operations. *Behav Res Methods Instrum Comput* **17**, 625-655 (1985).

6 Schmidt, C. *et al.* Homeostatic sleep pressure and responses to sustained attention in the suprachiasmatic area. *Science* **324**, 516-519 (2009)

Figure S1: Spectral power distribution ($W/m^2/nm$) of the polychromatic bright light source

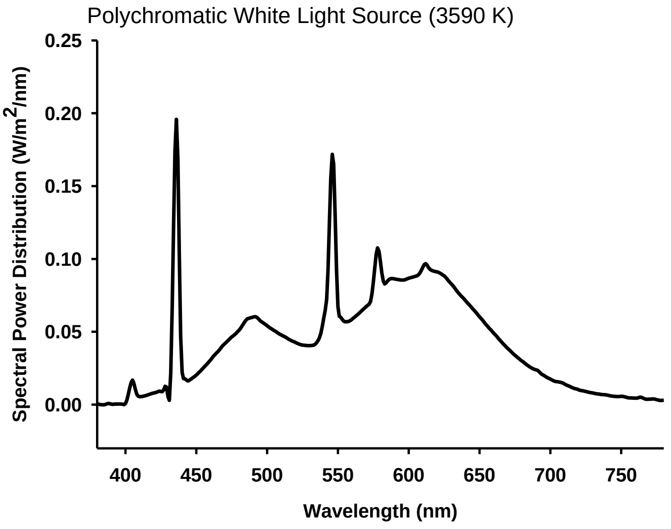

Figure S2: Effect sizes for pupil results (Cohen's d) for red and blue light pupil responses

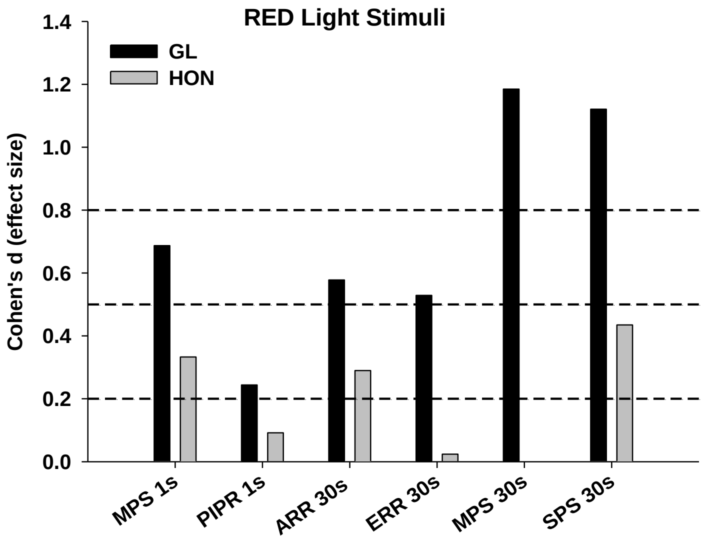

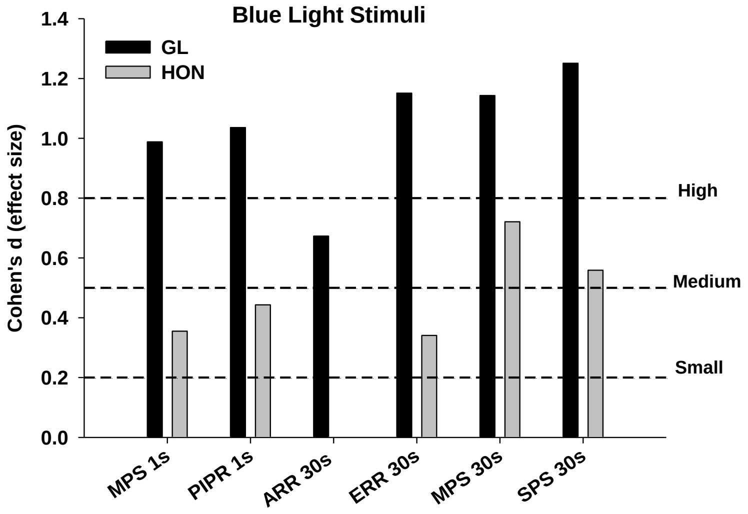

Supplement: Supplementary Information [file srep15185-s1.pdf]
